# Pseudopterosin Inhibits Proliferation and 3D Invasion in Triple-Negative Breast Cancer by Agonizing Glucocorticoid Receptor Alpha

**DOI:** 10.3390/molecules23081992

**Published:** 2018-08-10

**Authors:** Julia Sperlich, Nicole Teusch

**Affiliations:** Bio-Pharmaceutical Chemistry and Molecular Pharmacology, Faculty of Applied Natural Sciences, Technische Hochschule Koeln, Chempark, 51373 Leverkusen, Germany; julia.sperlich@th-koeln.de

**Keywords:** pseudopterosin, triple-negative breast cancer, glucocorticoid receptor alpha, dexamethasone, cell proliferation, 3D invasion, tumor spheroid, coculture, interleukin 6, interleukin 8

## Abstract

Pseudopterosin, produced by the sea whip of the genus *Antillogorgia*, possesses a variety of promising biological activities, including potent anti-inflammatory effects. However, few studies examined pseudopterosin in the treatment of cancer cells and, to our knowledge, the ability to inhibit triple-negative breast cancer (TNBC) proliferation or invasion has not been explored. Thus, we evaluated the as-yet unknown mechanism of action of pseudopterosin: Pseudopterosin was able to inhibit proliferation of TNBC. Interestingly, analyzing breast cancer cell proliferation after knocking down glucocorticoid receptor α (GRα) revealed that the antiproliferative effects of pseudopterosin were significantly inhibited when GRα expression was reduced. Furthermore, pseudopterosin inhibited the invasion of MDA-MB-231 3D tumor spheroids embedded in an extracellular-like matrix. Remarkably, the knockdown of GRα in 3D tumor spheroids revealed increased ability of cells to invade the surrounding matrix. In a coculture, encompassing peripheral blood mononuclear cells (PBMC) and MDA-MB-231 cells, and the production of interleukin 6 (IL-6) and interleukin 8 (IL-8) significantly increased compared to a monoculture. Notably, pseudopterosin indicated to block cytokine elevation, representing key players in tumor progression in the coculture. Thus, our results reveal pseudopterosin treatment as a potential novel approach in TNBC therapy.

## 1. Introduction

Breast cancer is still the most common malignancy in women with one million cases annually worldwide [1]. Of these, approximately 15% belongs to the triple-negative (ER^−^/PR^−^/HER2^−^) breast cancer (TNBC). TNBC represents the most aggressive breast cancer type, characterized by high proliferation rate, a pronounced potential to metastasize, and a shorter survival rate [2,3,4]. Furthermore, TNBC lacks effective therapies available for other breast cancer subtypes, underlining the significant unmet medical need for identifying novel targets and developing innovative drugs.

The tumor microenvironment is increasingly recognized as a major regulator of carcinogenesis. In breast cancer, tumor-associated macrophages (TAMs) enhance proliferation and metastasis as well as resistance to chemotherapy by activation of the transcription factor nuclear factor κB (NF-κB), a key factor in regulating inflammatory responses [5,6]. High expression levels of the NF-κB target genes interleukin 6 (IL-6) or interleukin 8 (IL-8) secreted by macrophages and can be correlated with advanced growth of TNBC and poor prognosis [7].

The pseudopterosins, a family of 31 known related diterpene glycosides, are produced by the sea whip *Antillogorgia elisabethae* (formerly named *Pseudopterosin elisabethae*) [8]. Striking biological activities have been described ranging from anti-inflammation [9,10,11], wound-healing [10,11], and analgesia-reducing [9,12,13] to neuromodulation [14]. In contrast, to date, little is known regarding the antitumor effects of pseudopterosin, where only one derivative showed moderate cytotoxic effects on ER^+^ breast cancer cells and non-small-cell lung cancer cells [15].

Previously, we have described the potential of pseudopterosin as a novel immune modulator in TNBC, acting via NF-κB inhibition and subsequent blockade of cytokine secretion [16]. Moreover, we identified the inhibitory capabilities of pseudopterosin on the NF-κB signaling pathway by agonizing the glucocorticoid receptor α (GRα) [16]. Accordingly, there is evidence that NF-κB and GRα can physically interact and heterodimerize in breast cancer [17]. By binding other transcription factors, such as NF-κB, GRα can either transactivate or suppress its target genes [18]. 

Although glucocorticoids (GCs) are frequently used to relieve symptoms of cancer treatment-related side effects, contradictory effects on breast cancer progression upon GC treatment and with respect to GRα expression have been described [19,20,21]. High expression levels of GRα in ER^−^ breast cancer might be associated with drug resistance, resulting in an unfavorable clinical outcome [22,23,24]. In contrast, a recent analysis demonstrates improved survival independent of the ER status in breast cancer patients receiving GC combined with adjuvant anthracycline-based chemotherapy [25]. Thus, in the current study we further elucidated the role of GRα in TNBC progression, thereby focusing on pseudopterosin as a novel agent for breast cancer therapy.

## 2. Results

### 2.1. Pseudopterosin Inhibited Proliferation of Triple Negative Breast Cancer Cells

**Figure 1:**
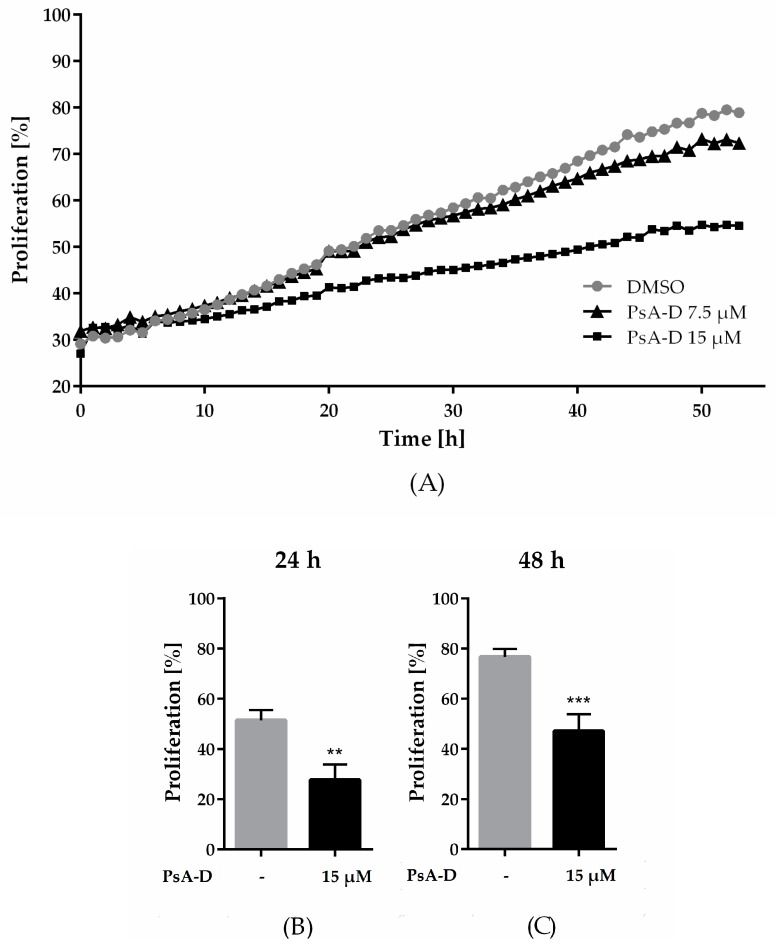
Pseudopterosin inhibited proliferation in triple-negative breast cancer cells. (**A**) Proliferating cells were imaged every hour over a time range of 50 h with the IncuCyte^®^ ZOOM. Confluency of cells was determined with IncuCyte^®^ software indicated as proliferation in percentage. Cells were treated with either 7.5 µM (triangle) or 15 µM (square) of pseudopterosin extract (PsA-D). (**B**,**C**) Inhibition of proliferation is shown at selected time points of 24 and 48 h compared to DMSO control, respectively. The data represent means of three independent experiments. Error bars were calculated using ±SEM. *p*-values were calculated against DMSO control. Two stars represent a significance of *p* < 0.01 and three stars represent a significance of *p* < 0.001.

In our previous work, we identified the natural product pseudopterosin as a novel inhibitor of NF-κB signaling [16], one key pathway in controlling progression of TNBC. As NF-κB is known to regulate various processes in cancer progression, such as proliferation, angiogenesis, or invasion [26,27,28], the aim of the current study was to further characterize the pharmacological properties of pseudopterosin. First, we investigated a pseudopterosin extract (PsA-D) regarding its effect on breast cancer cell proliferation in MDA-MB-231 cells. To remain within a nontoxic concentration range of PsA-D (IC_50_ values of cell viability for PsA-D after 24 h or 48 h of treatment were 31.4 µM and 32.2 µM, respectively; Supplemental Figure S1A,B), 7.5 and 15 µM of PsA-D were chosen to evaluate antiproliferative effects (Figure 1A). As expected, MDA-MB-231 cells treated with DMSO showed a high proliferation rate, represented by a confluency of 78% after 48 h (Figure 1A). Notably, a concentration of 15 µM of PsA-D was able to reduce proliferation significantly after 24 h by 1.9-fold and after 48 h by 1.6-fold compared to DMSO control (Figure 1B,C). Furthermore, preliminary data indicate that pseudopterosin-induced reduction of proliferation is not pERK dependent (Supplemental Figure S3), which is a key regulator for cell proliferation in principle [29].

### 2.2. GRα Expression is Essential for Antiproliferative Effects of Pseudopterosin

**Figure 2:**
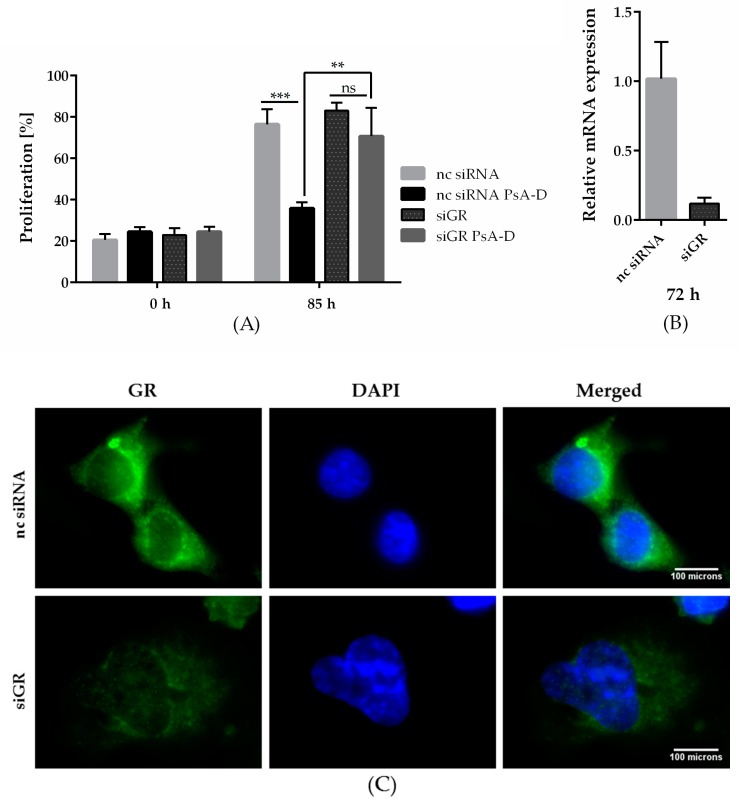

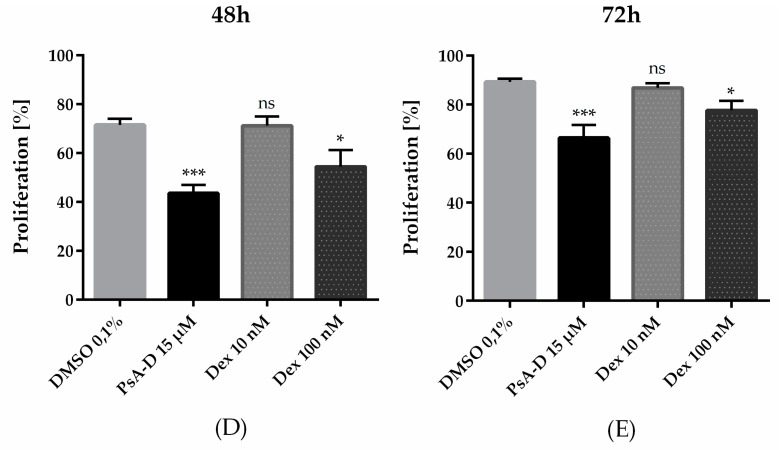
Pseudopterosin failed to inhibit breast cancer cell proliferation after knockdown of the glucocorticoid receptor alpha (GRα) and inhibited proliferation of MDA-MB-231 more efficaciously than dexamethasone (Dex). (**A**) Knockdown of GRα was done with the Lonza Nucleofector 2b device on day one. On day two, the cells were seeded and proliferating cells were imaged with the IncuCyte^®^ ZOOM every hour over a time range of five days. Cell proliferation was determined with IncuCyte^®^ software indicated in percentage. Cells were treated with a concentration of 15 µM of PsA-D. (**B**) After knockdown of GRα, expression of GRα reduced by up to 88.3%, which was confirmed by qPCR analysis at 72 h. (**C**) Immunofluorescent analysis of GRα knockdown after 72 h. Scale bars in white show 100 microns in length. (**D**,**E**) PsA-D inhibited proliferation after 48 and 72 h more efficaciously than Dex. The data represent means of three independent experiments. Error bars were calculated using ±SEM. One star represents a significance of *p* < 0.05, two stars of *p* < 0.01 and three stars of *p* < 0.001. “ns” means not significant.

In our previous work, we hypothesized pseudopterosin to act as an agonist of the GRα [16]. Subsequently, when downregulating GRα, pseudopterosin failed to inhibit NF-κB target gene expression. Thus, to further explore the role of GRα in the mode of action of pseudopterosin, we analyzed the effect of a GRα knockdown on breast-cancer-cell proliferation. After 72 and 85 h, treatment with PsA-D inhibited proliferation in noncoding siRNA (nc siRNA) transfected cells by 2-fold, respectively (Figure 2A and Supplementary Figure S4). Importantly, in siGRα transfected cells, PsA-D lost its antiproliferative effect (Figure 2A). Efficiency of the GRα knockdown using real-time qPCR (up to 88%) is exemplified in Figure 2B and depicted on the protein level via immunfluorescence analysis in Figure 2C. In conclusion, our data suggest that GRα expression might be crucial for the antiproliferative effects of PsA-D. 

Notably, treatment with the marked GRα ligand dexamethasone showed less potency in reducing proliferation: after 48 h, PsA-D resulted in a 21% proliferation decrease, whereas 100 nM dexamethasone reduced proliferation by 15% compared to DMSO, respectively (Figure 2C). After 72 h, PsA-D treatment diminished proliferation by 20%, whereas treatment with 100 nM dexamethasone reduced the proliferation rate by only 9% (Figure 2D).

### 2.3. Pseudopterosin Inhibited Invasion into 3D Matrix

**Figure 3:**
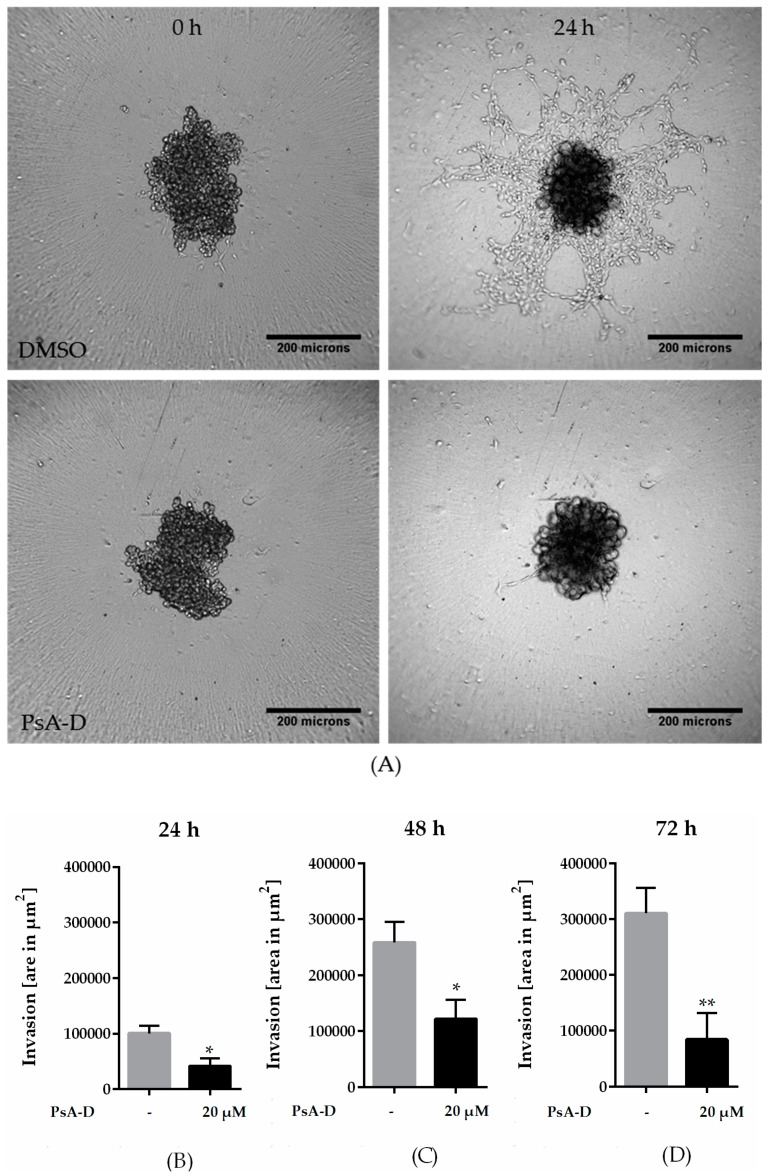
Pseudopterosin inhibited invasion into a 3D matrix. (**A**) Representative images of invasion of cells into a 3D matrix at the 24 h time point. Cells were imaged with IncuCyte^®^ ZOOM over a time range of three days. 3 × 10^3^ cells per well were seeded into ultra-low-attachment (ULA) round-bottom plates and spheroids were formed for 72 h. Scale bars in black show 200 microns in length. (**B**–**D**) The bar diagrams show three different time points representing six independent experiments. Spheroids were treated with a concentration of 20 µM of PsA-D. Error bars were calculated using ±SEM. *p*-values were calculated against control (CTRL). Two stars represent a significance of *p* < 0.01 and one star represents a significance of *p* < 0.05.

Breast tumors harbor many devastating characteristics resulting in poor prognosis of patients: high proliferation rate and high histological grade. Furthermore, genetic and epigenetic alterations enable breast cancer cells to migrate and invade the surrounding tissue via a process known as epithelial-to-mesenchymal transition (EMT) [30]. To explore the effects of pseudopterosin on the invasiveness of MDA-MB-231 cells, we developed a 3D invasion assay, where the cancer cells form a microtumor spheroid embedded in extracellular matrix (ECM). In the presence of DMSO, the cells immediately started to invade into the 3D matrix by partly disassembling the spheroid core (Figure 3A). In contrast, treatment with PsA-D significantly inhibited the invasion of single cells into the matrix. After 24 h, the invasive area was reduced significantly by 59%, after 48 h by 53%, and after 72 h by 73% (Figure 3B–D). Importantly, spheroid growth did not change after PsA-D treatment (Supplemental Figure S5). Thus, in our experiment we verified the inhibitory properties of pseudopterosin in a 3D assay on TNBC progression, thereby hinting at a better prediction for future in vivo tumor models with this natural product.

### 2.4. Downregulation of Glucocorticoid Receptor Alpha Expression Increased Invasiveness in TNBC

**Figure 4:**
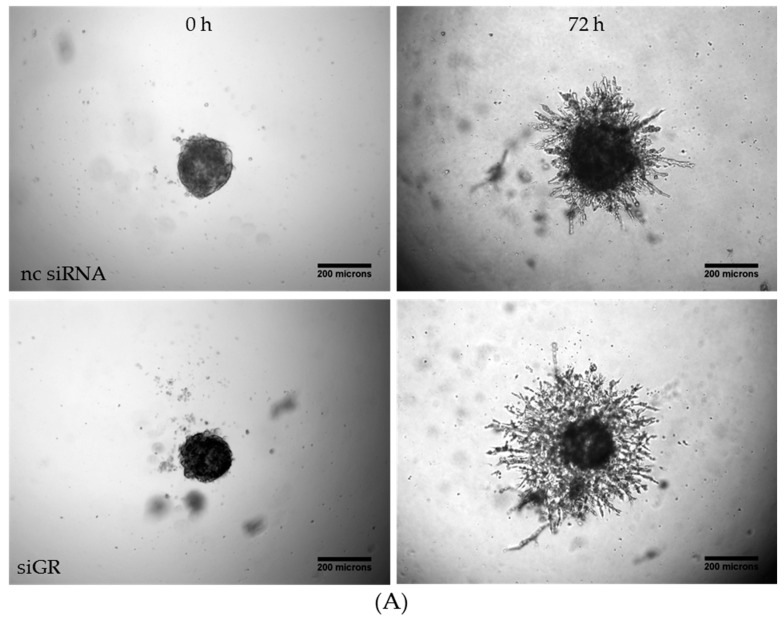

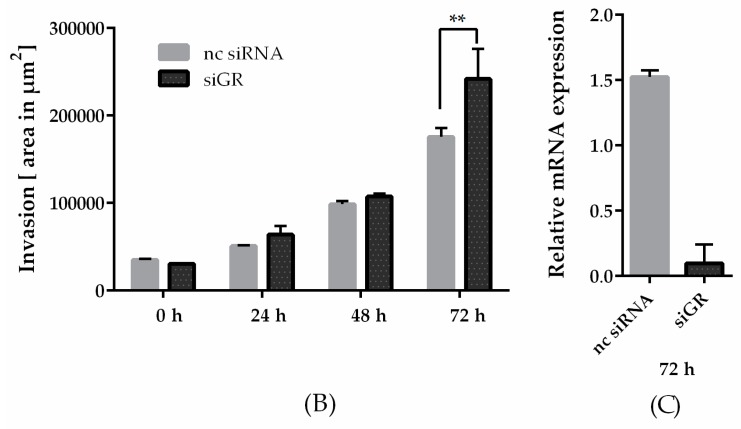
Knockdown of the GRα increased invasiveness of triple-negative breast cancer. (**A**) Representative images of tumor cell invasion into a 3D matrix. Knockdown of GRα was performed with the Lonza Nucleofector 2b device on day one. On day three, 3 × 10^3^ cells per well were seeded into ULA round-bottom plates. Formation of spheroids was allowed for 72 h. At t = 0, matrigel was added to the spheroids to start invasion. Scale bars in black show 200 microns in length. (**B**) The invasion is depicted over a time range of three days and the area of invaded cells into matrigel was calculated with ImageJ FIJI at the respective time points. (**C**) As confirmed by qPCR analysis, GRα expression is reduced up to 94% after 72 h. The data represent means of three independent experiments. Error bars were calculated using ±SEM. *p*-values were calculated against noncoding siRNA (nc siRNA) control. Two stars represent a significance of *p* < 0.01.

The clinical use of GCs is discussed controversially due to extensive side effects, chemotherapy resistance, and survival of cancer cells [21,23,31]. However, the recent literature indicates the beneficial effects of GCs to be strongly dependent on the tumor entity: survival in patients receiving GC combined with anthracycline-based chemotherapy was improved [25]. In this context, we further investigated the role of GRα in the invasiveness of MDA-MB-231 microtumor spheroids (Figure 4A). The efficiency in GRα knockdown is represented by a reduction of 94% (Figure 4C). After 72 h, the spheroids transfected with siGRα showed a significant increase in invasion by 27% compared to nc siRNA (Figure 3B). In conclusion, the knockdown of GRα led to an elevation of invasiveness in MDA-MB-231 cells, suggesting a potential of GRα agonists like pseudopterosin in diminishing TNBC progression.

### 2.5. Pseudopterosin Inhibited Cytokine Release in a Coculture of Primary Blood Mononuclear Cells (PBMC) and TNBC Cells

**Figure 5:**
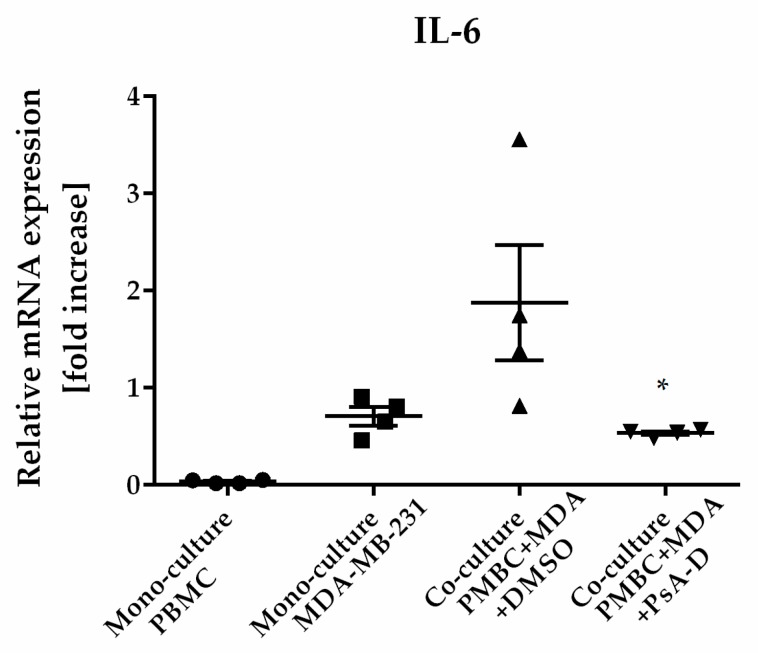
Pseudopterosin inhibited cytokine expression in a coculture of PBMC and MDA-MB-231. Both cell lines were cocultured at a ratio of 1:1 before treatment with 30 µM PsA-D. Cells were harvested 24 h after treatment and cytokine expression levels were analyzed with qPCR. Relative mRNA levels were normalized to fold increase. MDA is equivalent for MDA-MB-231 cells. Data represent means of four independent experiments. Standard deviation was calculated using ±SEM. *p*-values were calculated between ‘coculture’ and ‘coculture + PsA-D’ using Dunnett’s multiple-comparisons test. One star represents a significance of *p* < 0.05.

The microenvironment plays a critical role in breast cancer carcinogenesis [32]. TAMs are the drivers of breast-cancer-cell invasion [33,34]. A main characteristic of inflammatory breast cancer is the secretion of proinflammatory cytokines such as IL-6 or IL-8 by macrophages, regulating angiogenesis and promoting tumor progression [35,36]. Previously, we verified a blockade of NF-κB-dependent cytokine expression and secretion after pseudopterosin treatment in both MDA-MB-231 and THP-1 cells [16]. In this context, GRα knockdown led to the failure of pseudopterosin to inhibit cytokine expression. Furthermore, as shown previously, stimulation by the TLR4 ligand LPS leads to the production of cytokines and the subsequent secretion into the surrounding “conditioned medium” (CM) [16]. Our current data amend a significant reduction of cytokine expression, such as IL-6, IL-8 and TNFα, after PsA-D treatment in PBMC (Supplemental Figure S6). Medium containing cytokines released by MDA-MB-231 cells, representing the so-called “MDA-MB-231 CM” (M-CM), induced a significant cytokine expression in PBMC. Notably, pseudopterosin treatment was able to block cytokine expression induced by breast cancer cell-conditioned media in PBMC. (Supplemental Figure S6), which is not caused by toxicity of pseudopterosin on PBMC cells (Supplemental Figure S2). Thus, to further evaluate the pharmacological effects of pseudopterosin on bidirectional communication, we set up a coculture encompassing PBMC and MDA-MB-231 cells to analyze the change in IL-6 and IL-8 expression levels. In the coculture model, PsA-D treatment significantly inhibited IL-6 expression by 52.6% and IL-8 expression by 76.8%, respectively (Table 1). The fold increase of the IL-6 expression level in coculture increased by 1.9 compared to monoculture (Figure 5). As expected, PsA-D treatment reduced IL-6 expression levels by 3.5-fold (Figure 5). To further explore the agonism of pseudopterosin and GRα in the context of our coculture model, the focus in future studies will lay in continuing investigations concerning knockdown studies of GRα. Taken together, our data indicate that pseudopterosin has the potential to inhibit the proliferation, the invasiveness, and the communication of PBMC and MDA-MB-231 cells in a coculture model. Thereby, the inhibitory activity of pseudopterosin seems to depend on GRα expression. 

## 3. Discussion

For pseudopterosin, effective biological activities in various therapeutic areas, including anti-inflammatory effects, are described [9,10,11]. This study aimed to explore the inhibitory capabilities of pseudopterosin on distinct features of TNBC, namely the ability to invade surrounding tissue and the contribution to rapid tumor progression. For TNBC, a disease with a high unmet medical need and a low survival rate, we demonstrated previously a novel potential of pseudopterosin by inhibiting NF-κB signaling and subsequent cytokine secretion [16]. Furthermore, suggested by the translocation of GRα, we revealed a role of GRα activation upon pseudopterosin treatment. In the current study, GRα again indicated to play a role in mediating pseudopterosin-induced inhibition of breast-cancer-cell proliferation. 

Among others, NF-κB is an important regulator in the development of the mammary glands [37]. However, chronic inflammation in general and inflammation in the tumor microenvironment in particular, caused by NF-κB upregulation over a long time range, increases aggressiveness, invasivenes [38,39], and correlates with poor prognosis in breast-cancer patients [40]. As our data suggest pseudopterosin to inhibit constitutive NF-κB activity in TNBC cells [16], we further examined effects of pseudopterosin on blocking invasion. Adipocytes in breast tumors are described to secrete high amounts of collagen leading to increased tumor growth [41]. Despite using equivalently high collagen concentrations, which is known to reduce drug sensitivity [42], pseudopterosin displayed strong anti-invasive properties. Moreover, in a GRα knockdown, invasiveness in breast cancer tumor spheroids increased.

Gene expression analysis of breast tumors revealed a downregulation of genes involved in cell differentiation, whereas genes promoting tumorigenesis were upregulated [43]. However, mutations alone cannot explain the high malignancy and complexity of the tumor. The tumor microenvironment is the most important factor of why immune cells undergo a reprogramming step, thereby promoting tumor progression. The discovery that normal mammary epithelial cells cooperate with innate immune cells for invasive processes led to the discovery that macrophages are the drivers of intravasation from invasive breast tumors by establishing the tumor microenvironment [33,44]. Thereby, ECM, stromal cells such as endothelial and immune cells, fibroblasts, and adipocytes are the main components of the microenvironment [45]. Additionally, TAMs play a critical role in the tumor microenvironment by secreting second messengers such as IL-8 or IL-6 via NF-κB activation, thus promoting the tumor microenvironment and regulating angiogenesis, which, in turn, correlates with poor outcome and malignant features in breast cancer [35,36,46,47]. Paradoxically, cytotoxic chemotherapy further initiates TAM recruitment into invasive carcinoma [48], where coculture with breast cancer cells results in high IL-6 levels leading to the activation of cancer stem cells [49]. We confirmed elevated IL-6 and IL-8 expression levels as a result of cocultivating PBMC and MDA-MB-231 cells, where pseudopterosin was able to significantly block cytokine expression and henceforth the communication of both cell types.

In the clinics, GCs are used to reduce allergic reactions or nausea during chemotherapy due to upregulation of anti-inflammatory signals [50,51,52]. On tumor cells, the synthetic GRα ligand dexamethasone (Dex) has been described to reduce cell proliferation by decreasing ERK phosphorylation in ER^+^ breast cancer cells, possibly via the mechanism of transactivation [51]. ERK is a key regulator of proliferation and remodels the chromatin structure [29]. To our knowledge, antiproliferative effects of Dex were as yet not observed in MDA-MB-231 cells. In contrast, Dex was described to increase tumor growth and act as a proproliferative [53]. However, in our study, we not only observed antiproliferative effects after Dex treatment, but also witnessed improved antiproliferative effects of pseudopterosin treatment compared to Dex. Interestingly, preliminary data indicate that the mechanism of action of pseudopterosin seems to be distinct from Dex, as the phosphorylation status of ERK did not change in the presence of pseudopterosin. 

To date, GRα signaling can be divided into two distinct pathways: the so-called “transactivation”, reflecting target gene expression, and the “transrepression”, representing the downregulation of parallel signaling pathways, such as NF-κB activation. Prominent metabolic side effects of glucocorticoid treatment might be ascribed to transactivation of GRα [54]. In contrast, positive effects of GCs include reduced migration and a reduction in proteins associated with chemotherapy resistance in TNBC cells, which might be explained by transrepression of GRα [55,56,57]. The mechanism of the transrepressive process of GRα can have different origins: GRα can heterodimerize and bind directly to the p65/p50 dimer [58] or GRα recruits histone deacetylases to the promotors of inflammatory genes [59]. GRα transrepression is thereby defined as a direct interaction with transcription factors, for example NF-κB, without binding to DNA response elements and independent of IκB, p50, or p65 regulation of expression [54]. Thus, upregulation of IκBα expression [60] or repression of IL-8 by transcriptional inhibition of NF-κB are correlated with transactivation of GRα [54]. After GRα knockdown, we observed increased invasiveness in tumor spheroids and a lack of pseudopterosin to inhibit proliferation or invasion. Thus, we suggest the expression of GRα to be beneficial in maintaining a less invasive phenotype in TNBC and propose pseudopterosin to address the mechanism of transrepression by agonizing GRα.

In conclusion, we demonstrated the inhibitory effects of pseudopterosin on pronounced characteristics of TNBC, including tumor-cell proliferation and invasion. Our results imply pseudopterosin as a potential therapeutic basis suitable for targeting TNBC. Future studies will focus on investigating molecular function, including transrepressive effects of GRα in mediating pseudopterosin-dependent pharmacological actions. 

## 4. Materials and Methods 

### 4.1. Cell Culture and Reagents

The origin of the extract of pseudopterosin A to D isolated from *A. elisabethae* (subsequently named PsA-D) was kindly provided by Dr. Russell Kerr (University of Prince Edward Island, Marine Natural Products Lab, Canada) as described in our previous work [16]. U0126 inhibitor was purchased from Selleckchem (Houston, TX, USA). MDA-MB-231 breast-cancer cells were obtained from the European Collection of Authenticated Cell Cultures (ECACC, Salisbury, UK) and grown in humidified atmosphere containing 5% CO_2_ in an RPMI medium. Medium was supplemented with 15% FCS, 100 units·mL^−1^ penicillin, and 100 µg·mL^−1^ units streptomycin. PBMCs were purchased from STEMCELL Technologies (Vancouver, Canada) and cultured in the presence of 5% CO_2_ in RPMI along with 10% FCS, penicillin, and streptomycin. Staurosporin was purchased from Sigma-Aldrich (St. Louis, MO, USA) and medium and antibiotics from Life Technologies (Gibco, Carlsbad, CA, USA).

### 4.2. Real-Time Cell Proliferation 

MDA-MB-231 breast-cancer cells were seeded at a density of 1 × 10^5^ cells per mL in 96-well image-lock plates (Sartorius, Goettingen, Germany) and images were taken every hour for a time frame of five days with the IncuCyte^®^ Zoom from Sartorius (Goettingen, Germany). Confluency of cells was determined using the software of IncuCyte^®^ Zoom (Version 2016B).

### 4.3. Knockdown Studies 

GRα siRNA (siGRα) sc-35505 was purchased from Santa Cruz Biotechnology (Dallas, TX, USA). Silencer^®^ Select Negative Control No. 2 siRNA (nc siRNA) was obtained from Life Technologies (Carlsbad, CA, USA). 1 × 10^6^ cells were transfected with 300 nM siRNA using the Nucleofector 2b device (Lonza, Basel, Switzerland) using the X-013 protocol for transfection of MDA-MB-231 cells. After different time points, cells were harvested and expression upon knockdown of interest was analyzed using quantitative real-time PCR, respectively.

### 4.4. Quantitative Real-Time PCR

To determine cytokine or GRα expression levels after coculture or knockdown, the following primers were used (purchased from Eurofins, Ebersberg, Germany): IL-6 forward (GGCACTGGCAGAAAACAACC), IL-6 reverse (GCAAGTCTCCTCATTGAATCC), IL-8 forward: (ACTGAGAGTGATTGAGAGTGGAC), IL-8 reverse: (AACCCTCTGCACCCAGTTTTC), GAPDH forward: (TGCACCACCAACTGCTTAGC), GAPDH reverse: (GGCATGGACTGTGGTCATGAG), GR forward: (AAAAGAGCAGTGGAAGGACAGCAC), GR reverse: (GGTAGGGGTGAGTTGTGGTAACG). Total RNA was isolated with “RNase Mini kit” from QIAGEN (Hilden, Germany) according to the manufacturer’s instructions and reverse transcriptase PCR was performed using “Reverse Transcription Kit” from Promega (Darmstadt, Germany). Real-time PCR was conducted with “Quantitect SYBR Green” from QIAGEN based on the following protocol: Preincubation at 95° for 900 s, amplification was performed over 45 cycles (95° for 15 s, 55° for 25 s, and 72° for 10 s). Nontemplate controls served as negative controls. C_T_ values were calculated according to the 2^−ΔΔC^_T_ method [61]. Sample values were normalized to the housekeeping gene GAPDH (glyceraldehyde 3-phosphate dehydrogenase).

### 4.5. 3D Invasion Assay

To study MDA-MB-231 invasion into an ECM such as matrigel (Corning, New York, NY, USA), spheroids of MDA-MB-231 were generated for 72 h starting with 3 × 10^3^ cells and 0.25% matrigel in an ultra-low-attachment (ULA) plate (Corning, New York, NY, USA). Invasion was initiated by addition of matrigel in a ratio of 1:1 volume to the spheroids. Images were taken with the IncuCyte^®^ Zoom (Sartorius, Goettingen, Germany), to create a time-lapse movie, or the Axio Vert.A1 microscope (Zeiss, Oberkochen, Germany) every 24 h for a time frame of 3 days. Image analysis was done with ImageJ makro “Analyze Spheroid Cell Invasion in 3D matrix” by Volker Baecker [62] (FIJI distribution [63]).

### 4.6. Coculture Studies

Coculture of PBMC and MDA-MB-231 cells: PBMC were freshly thawed for each experiment. 1 × 10^6^ cells of MDA-MB-231 were seeded on day 1 and incubated with PsA-D for 20 min on day 2. Treatment was followed by addition of PBMC cells to the MDA-MB-231 cells at a ratio of 1:1. Finally, cells were harvested at day 3 and analyzed for cytokine expression by real-time PCR.

### 4.7. Preparation of PsA-D Mixture

*A. elisabethae* was collected from South Bimini Island, as described in our previous work [16]: the extract was dried and extracted in EtOAc/MeOH (1:1) for 48 h and subjected to silica-gel chromatography eluting with hexanes and EtOAc to afford a mixture of PsA-D. The ratio was determined to be 85:5:5:5 (PsA:B:C:D) by LC-MS analysis.

### 4.8. Immunofluorescent Staining

After treatment according to Section 4.3, cells were fixed with –10 °C cold methanol for 5 min and treated with 0.1% Triton™ X-100 for 15 min. Antibodies were purchased from Santa Cruz Biotechnology (Dallas, TX, USA): primary antibody (sc-8992 GR (H-300)) incubated 1:50 for 24 h overnight at 4 °C and secondary antibody (sc-2012 IgG-FITC (fluorescein isothiocyanate)) was incubated 1:100 for 2.5 h at room temperature. For staining, the cell nuclei 4′,6-Diamidin-2-phenylindol (DAPI, Sigma Aldrich, St. Louis, MO, USA) were incubated for 5 min at room temperature at a concentration of 3 µM. Cells were washed 3 times with PBS following each incubation step. 

### 4.9. Statistical Analysis

All data shown represent at least 3 independent experiments. Error bars show ±SEM of all the means of triplicate values. Figures and statistical analysis were generated with Graphpad Prism v. 6.07 (Graphpad Software, San Diego, CA, USA) using one-way ANOVA and the underlying Dunnett’s multiple comparisons test. *p* < 0.05 was chosen to define statistically significant differences.

## Figures and Tables

**Table 1:** Inhibition of cytokine expression in coculture of peripheral blood mononuclear cells (PBMC) and MDA-MB-231 cells after pseudopterosin treatment.

	Monoculture PBMC	Monoculture MDA *	Coculture PBMC + MDA + DMSO	Coculture PBMC + MDA + PsA-D	*p*-Value ^1^
**IL-6 ^2^**	1.09 (± 3.2)	31.7 (± 20.3)	44.6 (± 25.3)	21.2 (± 12.7)	0.02
**IL-8 ^2^**	27.1 (± 36.9)	67.9 (± 46.5)	213.9 (± 99.6)	49.5 (± 13.2)	0.22

^1^*p*-values were calculated with ONE-Way ANOVA between ‘coculture’ and ‘coculture + PsA-D’. ^2^ The data represent relative mRNA expression values measured with real-time qPCR; * MDA is equivalent for MDA-MB-231 cells.

## Supplementary Materials

The following are available online, Figure S1: Cell Viability of MDA-MB-231 cells after pseudopterosin treatment. Figure S2: Cell viability assessment of PBMC cells after pseudopterosin treatment. Figure S3: Pseudopterosin did not change ERK phosphorylation status in MDA-MB-231 cells. Figure S4: Pseudopterosin failed to inhibit breast cancer cell proliferation after knockdown of the GRα after 72 h. Figure S5: Pseudopterosin does not inhibit spheroid growth. Figure S6: Pseudopterosin inhibited bidirectional communication between TNBC cells and PBMC.
