# Supplementary material for: Pseudopterosin Inhibits Proliferation and 3D Invasion in Triple-Negative Breast Cancer by Agonizing Glucocorticoid Receptor Alpha
Source: Molecules. 2018 Aug 10;23(8):1992. doi: 10.3390/molecules23081992 (PMC6222322; doi:10.3390/molecules23081992)
Supplement: Supplementary file 1 [file molecules-23-01992-s001.pdf]

molecules

MDPI

Supporting Information

Pseudopterosin Inhibits Proliferation and 3D Invasion in Triple-Negative Breast Cancer by Agonizing Glucocorticoid Receptor Alpha

Julia Sperlich and Nicole Teusch *

Biopharmaceutical Chemistry & Molecular Pharmacology, Faculty of Applied Natural Sciences, Technische Hochschule Koeln, Chempark, 51373 Leverkusen, Germany

* Correspondence: nicole.teusch@th-koeln.de; Tel.: +49-214-32831 (ext. 4623)

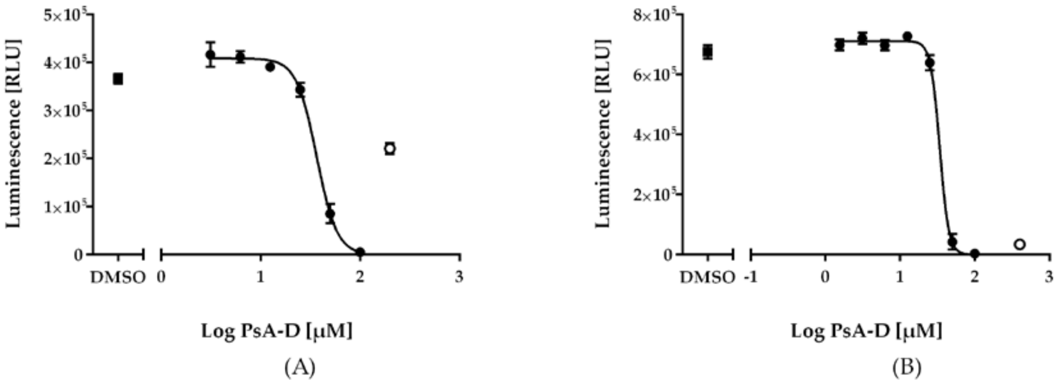

S1. Cell Viability of MDA-MB-231 cells after pseudopterosin treatment. Increasing amounts of PsA-D were incubated for either 24 hours showing an IC_{50} value of 31.4 µM (**A**) or for 48 hours leading to an IC_{50} value of 32.16 µM (**B**). Staurosporine (white circles) serves as positive control and 1% DMSO as negative control. Error bars were calculated using ±SEM. Graphs represent exemplary data. Means of IC_{50} values were calculated of three independent experiments.

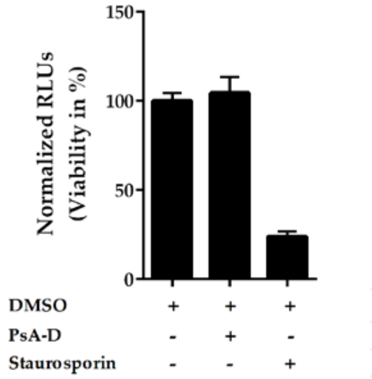

S2. Cell viability assessment of PBMC cells after pseudopterosin treatment. 30 µM of PsA-D were tested for its cytotoxic properties after 24 hours of treatment on PBMC cells. 3 µM staurosporine served as positive control and DMSO as negative control. Error bars were calculated using ±SEM. Graphs represents means of two independent biological repeats. RLU represents "Relative Luminescent Units".

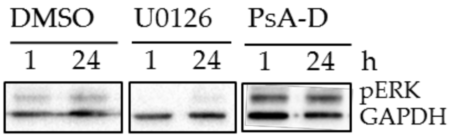

S3. Pseudopterosin did not change ERK phosphorylation status in MDA-MB-231 cells. Cells were treated with 15 µM of PsA-D and incubated for either 1 or 24 hours. DMSO served as negative control and the MEK inhibitor U0126 at a concentration of 10 µM as positive control. At the indicated time points, cells were harvested and 20 mg of protein were used for a western blot analysis. The housekeeping gene GAPDH served as a loading control.

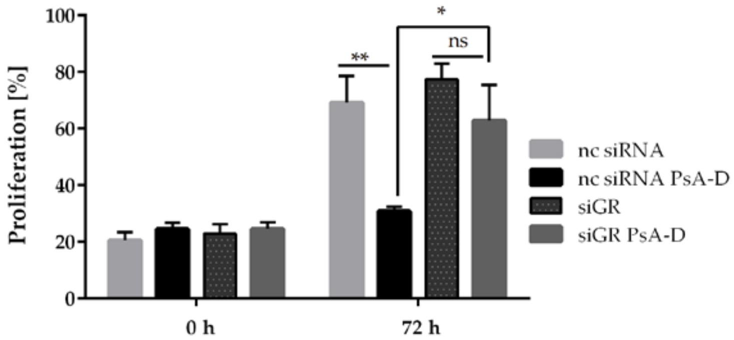

S4. Pseudopterosin failed to inhibit breast cancer cell proliferation after knockdown of the glucocorticoid receptor alpha (GRα) after 72 hours. Knockdown of GRα was done with the Lonza Nucleofector 2b device. The cells were seeded and proliferating cells were imaged with the IncuCyte® ZOOM every hour. Confluency of cells was determined with IncuCyte® software indicated in proliferation in percent. Cells were treated with a concentration of 15 µM of PsA-D. The bar diagram shows the proliferation rate at time points 0 and 72 hours. The data represent means of three independent experiments. Error bars were calculated using ±SEM. Two stars represent a significance of $p < 0.01$ and one star of $p < 0.05$.

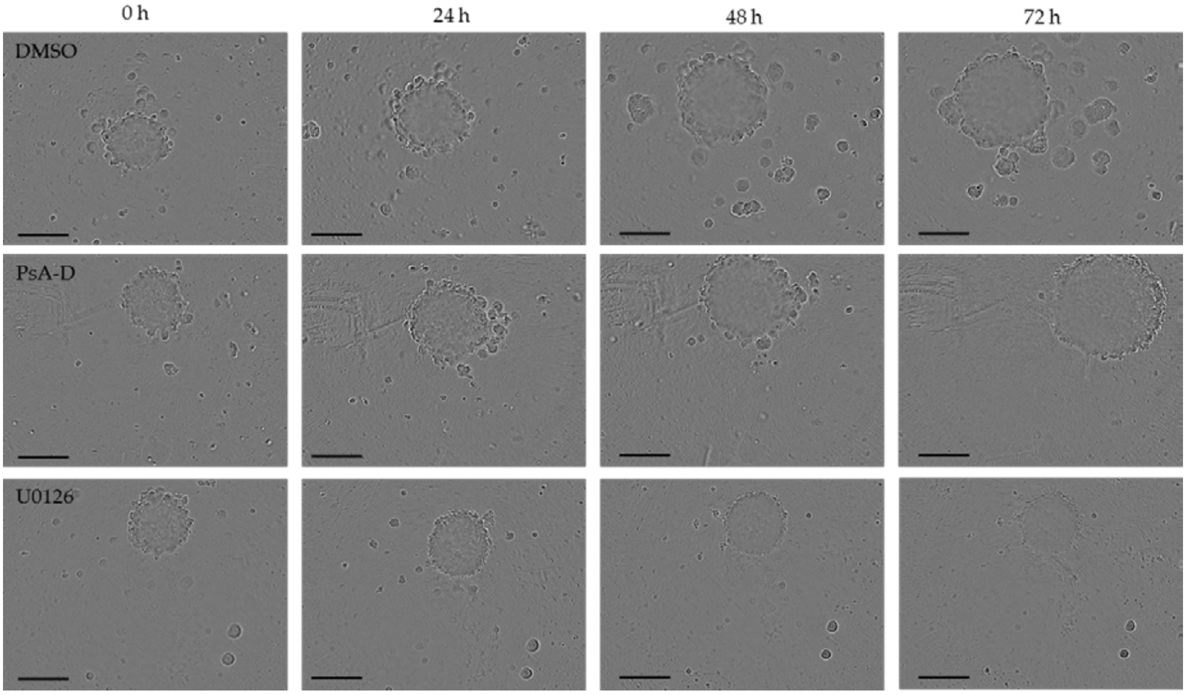

(A)

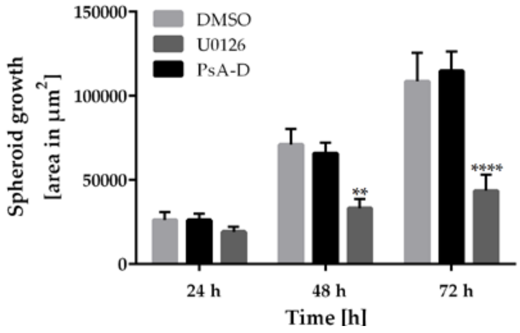

(B)

S5. Pseudopterosin did not inhibit spheroid growth. Spheroids were formed for 72 hours using $3*10^3$ cells and 2.5% matrigel. The spheroids were imaged with the IncuCyte® ZOOM every hour for a time frame of three days. The growth of the spheroids was measured using Fiji ImageJ. As positive control, MEK inhibitor U0126 was added at a concentration of 10 µM and DMSO served as a negative control. PsA-D was added at a concentration of 30 µM. The data represent means of six independent experiments. Scale bars in black represent 300 microns. Error bars were calculated using ±SEM. Four stars represent a significance of $p < 0.0001$ and two stars of $p < 0.01$.

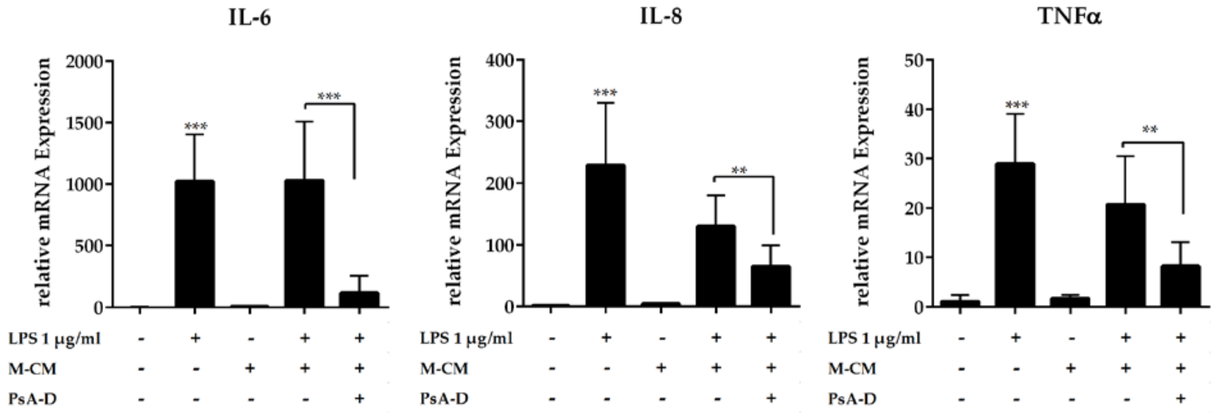

S6. Pseudopterosin inhibited bidirectional communication between triple-negative breast cancer (TNBC) and PBMC. Tumor-conditioned medium of MDA-MB-231 cells was produced using 1 µg/ml LPS and $1*10^6$ cells per ml. After 24 hours of incubation, tumor-conditioned medium (MDA-MB-231-conditioned medium, M-CM) was harvested, centrifuged, and sterile filtered. Afterwards, M-CM was added at 25-volume percentages to $1*10^6$ cells per ml of PBMC. After 5 hours of incubation, cells were harvested, RNA isolated, and analyzed with qPCR.

Supplementary Method 1: Measurement of cell viability: MDA-MB-231 cells were seeded at a density of $2.8*10^5$ cells per ml in 384 well plates (Greiner Bio-One, Kremsmuenster, Austria) with the CyBio pipetting robot (Analytic Jena AG, Jena, Germany) and PBMCs were seeded at a density of $1*10^6$ cells per ml in 96-well plates (Greiner Bio-One, Kremsmuenster, Austria). MDA-MB-231 cells were incubated for 24 hours at 37°C before treatment and PBMCs for one hour before treatment. Compounds were added at different concentrations and incubated either 24 or 48 hours, respectively. Measurement of cell viability was performed with CellTiterGlo® from Promega (Darmstadt, Germany) according to the manufacturer's instructions.

Supplementary Method 2: Western blot measurement of phosphorylated ERK in MDA-MB-231 cells: $1*10^6$ cells per ml were seeded into 6-well plates (Thermo Fisher Scientific, Waltham, USA) and

incubated for 24 hours at 37°C before treatment. After treatment, cells were harvested, lysed (5x Lysis buffer, Promega, Darmstadt, Germany) with a buffer containing protease and phosphatase inhibitors (Roche, Basel, Switzerland) and protein concentration of samples, determined with Roti®-Quant reagent (Roth, Karlsruhe, Germany), was adjusted to 20 mg. Samples were loaded on 12% SDS gels, run at 100 V, and afterwards blotted on a PVDF membrane at 25 V using a semidry installation (Bio-Rad Laboratories, Hercules, USA). The membrane was blocked with 5% nonfat dry milk (Roth, Karlsruhe, Germany). The housekeeping gene GAPDH was used as a loading control. Primary antibodies (GAPDH rabbit: D16H11; pERK rabbit: D13.14.4E) were purchased from Cell Signaling Technology (Danvers, USA), used at a dilution of 1/1000 in 5% nonfat dry milk and incubated over night at 4°C. The secondary antirabbit HRP-linked antibody (Cell Signaling Technology, Danvers, USA) was used at a dilution of 1/2000 and incubated for 2 hours at room temperature.

Supplementary Method 3: Spheroids of MDA-MB-231 cells were generated for 72 hours starting with $3*10^3$ cells and 2.5% matrigel (Corning, New York, U.S.) in an ultra-low-attachment (ULA) plate (Corning, New York, U.S.). U0126 MEK inhibitor (Sellekchem, Houston, U.S.) served as positive control. Images were taken with the IncuCyte® Zoom (Sartorius, Goettingen, Germany) every hour for a time frame of three days. Image analysis was done with ImageJ, FIJI distribution[63].

Supplementary Method 4: Production of conditioned medium (CM) from MDA-MB-231 cells: MDA-MB-231 cells were seeded at a density of 1×10^6 cells into a 25 cm^2 flask. Cells were either stimulated with 1 µg/mL LPS or without, serving as a negative control. MDA-MB-231 conditioned media (M-CM) was collected after 24 hours, centrifuged and sterile filtered. PBMCs were freshly thawed and seeded at 1×10^6 cells per ml. PsA-D was added at a concentration of 30 µM for 20 minutes followed by addition of 25 volume percentage of M-CM for 5 hours. Cells were then harvested and RNA isolated with RNase Mini Kit (Qiagen, Hilden, Germany) for further quantitative real-time PCR analysis.
